# Attachment to inanimate objects and early childcare: A twin study

**DOI:** 10.3389/fpsyg.2014.00486

**Published:** 2014-05-22

**Authors:** Keren Fortuna, Liora Baor, Salomon Israel, Adi Abadi, Ariel Knafo

**Affiliations:** ^1^Department of Psychology, The Hebrew University of JerusalemJerusalem, Israel; ^2^Ashkelon Academic CollegeAshkelon, Israel

**Keywords:** childcare, day care, object attachment, transitional object, LIST

## Abstract

Extensive non-maternal childcare plays an important role in children’s development. This study examined a potential coping mechanism for dealing with daily separation from caregivers involved in childcare experience – children’s development of attachments toward inanimate objects. We employed the twin design to estimate relative environmental and genetic contributions to the presence of object attachment, and assess whether childcare explains some of the environmental variation in this developmental phenomenon. Mothers reported about 1122 3-year-old twin pairs. Variation in object attachment was accounted for by heritability (48%) and shared environment (48%), with childcare quantity accounting for 2.2% of the shared environment effect. Children who spent half-days in childcare were significantly less likely to attach to objects relative to children who attended full-day childcare.

## INTRODUCTION

A growing number of children in the Western world routinely attend organized group-based childcare, and thus spend much of their days under non-maternal supervision and care. There is an ongoing debate regarding the implications of this shift from in-home to out-of-the-home care early in children’s lives (e.g., Belsky, 1986, 2001; Phillips et al., 1987; Jaffee et al., 2011), though it seems clear that non-maternal care indeed plays a role in children’s development. For example, high quality childcare has been linked with enhanced cognitive and academic functioning (McCartney, 1984; Caspi et al., 2000; NICHD Early Child Care Research Network, 2000). Childcare quantity (e.g., hours spent in daycare) has been shown to relate to behaviors such as aggression, non-compliance, and other externalizing problems (e.g., NICHD Early Child Care Research Network, 2000, 2010). Several studies also find early and extensive childcare to be related to increased risk for insecurity of young children’s attachment to their mothers (provided that maternal sensitivity is also low; NICHD Early Child Care Research Network, 1997).

Children’s center-based childcare experience has also been shown to relate to atypical diurnal rhythms of cortisol (a stress-related hormone) production (e.g., Dettling et al., 2000), pointing to it being a stressor for young children, perhaps due to having to deal with early separation from their attachment figures and the familiarity of the home environment, constant peer interactions, and limited focused adult attention. Given that, parents, daycare providers, and children themselves use various means of providing the child with a sense of security and continuity. A well-known, yet under-studied, developmental phenomenon in young children is their tendency to become strongly attached to inanimate objects, usually soft, cuddly toys, or blankets. Children’s use of objects is especially noted when children are under stressful situations or vulnerable states, such as in unfamiliar environments, when upset, ill, and tired. The favored objects, often referred to as “security blankets,” come to serve a comforting, anxiety-reducing function for the child.

The role of security provided by the attachment object has been demonstrated in a set of experimental manipulations (Passman and Weisberg, 1975; Passman, 1976, 1977). In a novel play setting, children exposed to their attachment object played and explored without evidencing distress for the same period of time as children who had their mothers in the room, and longer than children who were not attached to the present object. Thus, the presence of the child’s special object may have an arousal reducing effect while simultaneously facilitating exploration in a novel, moderately stressful situation. Of course, Harlow’s earlier experiments with infant monkeys showed similar patterns of fear-reduction and exploration in the presence of an inanimate cloth surrogate “mother” when monkeys were introduced to fear-producing stimuli (Harlow and Zimmermann, 1959). Object attachment can be thought of as constituting a protective factor (Rutter, 1985); although this inanimate object cannot provide reassurance, guidance or affection, its presence provides the child with a sense of protection. In the words of Harlow and Suomi (1970, p. 161): “Even though the cloth mother was inanimate, it was able to impart to its infant such emotional security that the infant would, in the surrogate’s presence, explore a strange situation and manipulate available physical objects.”

Despite some notions regarding the nature and functions that object attachments serve for young children, the sources for this developmental phenomenon have not been widely empirically studied, leaving the factors that contribute to children’s use of non-social objects for comfort not well understood. In order to better understand the basis for what appears to be an important coping mechanism for some children (but not others), this study, as a first step, employs a genetically sensitive design to assess the relative contributions of the environment and heritable factors to the development of individual differences in this behavior.

In addition, we hypothesized that placement in childcare centers functions as a specific environmental factor which contributes to the development of attachment to inanimate objects, perhaps as a mean for coping with early separation from attachment figures and other related stressors. Therefore, in the current study we examined whether non-maternal childcare is linked with children’s tendency to develop object attachments. Moreover, extending the classic twin design to include a measured environment component (e.g., Caspi et al., 2000; Jaffee et al., 2002), we also estimated the extent to which childcare accounted for variation in object attachment beyond latent genetic and environmental effects.

### OBJECT ATTACHMENT – PREVIOUS STUDIES

The theorist most often associated with acknowledging these behaviors in children is Winnicott (1953), who coined the term “transitional object” for a child’s treasured object. He emphasized that the use of transitional objects is common and considered it part of normal, healthy, development. The few empirical studies and surveys available on this developmental phenomenon, however, suggest that attachment to objects is not universal. In Western countries object attachments were indeed found to be common (e.g., van Ijzendoorn et al., 1983), with rates reaching as high as 60% (Passman and Halonen, 1979; Litt, 1981; Lehman et al., 1992). However, in other cultures, particularly those in which young children spend much of their time, both night and day, in close proximity to their mothers, rates of object attachments were found to be significantly lower (Gaddini and Gaddini, 1970; Hong and Townes, 1976; Litt, 1981). Attachment to inanimate objects has therefore been hypothesized to develop as an adaptation to child-rearing practices, such as amount of physical contact, sleeping arrangements, and the extent to which children need to cope with frequent separations from their mothers.

Indeed, originally Winnicott (1953) theorized that transitional objects help children manage the stress of separation from the mother by creating a symbol of her. Bowlby (1969/1982) described children’s treatment of their favored object as a substitute for their “natural” attachment figure when the person that is typically relied upon for comfort in anxiety-inducing situations is temporarily unavailable. Some support for this exists, demonstrating that for some children the emotional tie they develop to their “blankies” appears to reduce anxiety around separation experiences from their caregivers and facilitates smooth separations (Passman, 1977). For example, children were observed to use their attachment objects as they separated from caregivers at childcare (Triebenbacher and Tegano, 1993). In another study, children who were described by their mothers as independent and to have few difficulties going to bed (a form of separation) had significantly higher rates of object attachment than dependent children and those with sleep problems (Boniface and Graham, 1979).

### THE CURRENT STUDY

This is the first study to investigate the relative contribution of genetic and environmental influences to the development of object attachment, a notable developmental phenomenon. Using maternal reports – shown to be a valid measure of assessing object attachment (Weisberg and Russell, 1971) – we attained a large sample of twins at the age of three. Twin studies, the most widely used method for estimating genetic and environmental effects (Plomin et al., 2001), rely on a comparison between the similarity of monozygotic (MZ) twins, who are virtually 100% identical, and dizygotic (DZ) twins, who share on average half of the genetic variability. Greater similarity of MZ as compared with DZ twins indicates genetic influence (*heritability*). Similarity beyond this genetic effect is attributed to the environment that twins have in common (*shared environment* effect), and any further differences between twins are attributed to *non-shared environment* and measurement error.

Second, we focused on non-maternal childcare as a potential environmental contributor to children’s development of object attachment. We hypothesized that length of time spent daily in daycare centers would be related to higher rates of object attachment, given the long separation from the child’s main caregivers. Structural features of childcare quality, such as child-caregiver ratio and group size, were also examined in relation to object attachment. To our knowledge no study has explicitly examined these questions, although a couple of small-scale studies reported no associations between rates of object attachment and attending daycare, number of hours per week spent in non-parental care (Steir and Lehman, 2000), attending a play group, and hospitalization (Boniface and Graham, 1979). We address these issues in the largest study of children’s object attachment.

## MATERIALS AND METHODS

### PARTICIPANTS

#### Twins

Families with twins were contacted with surveys as part of The Longitudinal Israeli Study of Twins (LIST; Knafo, 2006; Avinun and Knafo, 2013). Contact details were provided by the local government office, based on information about all mothers giving birth to more than one child within 24 h during the years 2004–2005. As part of a comprehensive survey about children’s behavior and development mothers reported about their twins’ daycare setting and object attachment. The sample for the current report was comprised of 1122 pairs of twins. Children’s mean age at the time the surveys were completed was 36.86 months (SD = 2.14). Mean maternal age was 34.32 years (SD = 5.38). Family income, rated on a 5-point scale (*1* = a lot below average, *5* = a lot higher than average) was slightly above the midpoint of the scale (*M* = 3.15, SD = 1.35), and mothers completed between 6 and 25 years of education (*M* = 15.24, SD = 2.59).

Zygosity was determined by the Zygosity Questionnaire for Young Twins (Goldsmith, 1991) using an algorithm which has been shown to be over 95% accurate when compared to DNA testing (Price et al., 2000). This questionnaire asks regarding genetically determined physical differences between the twins (e.g., height, weight, hair and eye color, timing of teeth eruption), likelihood of confusion between them, and medical information (e.g., blood type, possible medical reasons for differences). The sample consisted of 217 MZ pairs (120 males, 97 females), 432 same-sex DZ (SSDZ) pairs (210 males, 222 females), and 398 opposite-sex DZ (OSDZ) pairs. Zygosity could not be determined for 75 same-sex pairs.

### MEASURES

#### Object attachment

Mothers were asked: “Does your child have an object (e.g., a toy, a blanket) from which she/he finds it hard to part, and carries it almost everywhere?” Possible responses were: “yes,” “yes, in the past,” and “no, never had.” If mothers indicated yes, they were asked to describe the object. Children were divided into two groups: children who had never developed object attachments and children who, at any point, had been attached to an object (i.e., we combined the options “yes,” and “yes, in the past”). Pacifier use, bottle use, and thumb sucking were not considered object attachments.

For 508 twin pairs information regarding object attachment was collected at age 3 (*M* = 36.94 months, SD = 1.97), and for 485 pairs at an age 5 assessment (*M* = 61.21 months, SD = 2.25). Among 129 twin pairs object attachment was reported at both time points, and there was good consistency in maternal reports from age 3 to 5 [*χ*^2^(1) = 46.59, *p* < 0.001]. In addition, only five children were reported to have an attachment object at age 5 and not at age 3, suggesting that time of reporting makes little difference. This resonates with the finding that object attachment develops mainly in the period before 36 months of age (Passman, 1987). Therefore, responses were combined, such that a child was considered to have an object attachment if the mother indicated so at either assessment.

#### Childcare

Mothers reported about the children’s current childcare arrangement: whether or not they attended an out-of-the-home daycare center, until what time each day, number of children in the group with their child, and number of daycare providers.

#### Risk at birth

Twins are at a higher risk for being born preterm (average gestational age at twin delivery is 36 weeks) and at a lower birth weight than singletons (e.g., Dollberg et al., 2005). Such factors are influential in children’s early development (e.g., Clark et al., 2008). We therefore asked mothers to report about their twins’ gestational age and weight at birth, and whether or not the child had been admitted to the Neonatal Intensive Care Unit (NICU), as detailed by Fortuna et al. (2011), to be able to control for risk in the analyses.

## RESULTS

Due to the non-independence of scores among twins within pairs, information regarding only one randomly chosen twin per pair (50% females) was used in all descriptive analyses and group comparisons.

### PREVALENCE AND TYPES OF OBJECT ATTACHMENTS

A third of the current sample (33%) was reported to have developed an attachment to inanimate objects. Of these, 38% were attached to a soft fabric (a rag, a piece of cloth, most often a cloth diaper), 19% had a blanket, 31% were attached to a soft doll or a teddy bear, 5% were using a pillow, and the remaining 7% were using other objects (e.g., hard toys).

There were no differences between boys and girls in rates of object attachment, *χ*^2^(1) = 0.51, ns, and types of objects used, *χ*^2^(4) = 4.25, ns. Likewise, zygosity groups did not differ on prevalence of object attachment, *χ*^2^(2) = 2.55, ns, and object type, *χ*^2^(8) = 3.79, ns. In addition, there was no difference in mean family income between the group of children who had an object attachment (*M* = 3.25, SD = 1.31) and children who were not object-attached (*M* = 3.10, SD = 1.34), *t*(982) = 1.62, ns, nor was there a difference in years of maternal education (*M* = 15.21, SD = 2.48 and *M* = 15.26, SD = 2.65, respectively), *t*(1045) = 0.05, ns.

### ENVIRONMENTAL AND GENETIC EFFECTS ON THE DEVELOPMENT OF OBJECT ATTACHMENT

In order to estimate rates of genetic and environmental influences on the development of object attachment, we compared MZ and DZ twin similarities. As the variable assessing object attachment is dichotomous (i.e., yes or no), we assessed proportions of twin pairs who were concordant on attachment object across zygosity groups, using probandwise concordance (a measure of the proportion of twins who exhibit the behavior, who have a twin who also exhibits that behavior). MZ twin pairs were concordant at 0.87 (i.e., in 87% of pairs in which one twin was attached to an object, the other twin was attached to an object as well). Among SSDZ pairs, concordance rate was only 0.69, and 0.62 for OSDZ. Thus, MZ twins were more likely to be concordant than were DZ twins, pointing to genetic influences on the development of individual differences in object attachment. Yet, similarity among DZ twins which is larger than half that of MZ twins points to a substantial shared environmental effect. In order to test whether neonatal risk factors moderate this effect we excluded from the analysis twins who were born at any of the following risks – prior to 36 week gestation, birth weight below the 10th percentile relative to comparable population norms, or were administered to the NICU—and found concordance rates to be similar (**Table 1**).

**Table 1:** Twin probandwise concordances on object attachment by zygosity.

	*N* pairs	MZ	SSDZ	OSDZ
Full sample	1035	0.87	0.69	0.62
Known neonatal risk	357	0.88	0.74	0.62
No known neonatal risk	585	0.88	0.68	0.63

*MZ, Monozygotic twins; SSDZ, same-sex dizygotic twins; OSDZ, opposite-sex dizygotic twins. Known neonatal risk = birth at less than 36 week gestation, low birth weight (below the 10th percentile), or admission to the Neonatal Intensive Care Unit.*

A more direct test of the estimates of the variance in object attachment accounted for by latent additive genetic, shared environmental, and non-shared environmental factors is done by conducting model fitting in structural equation modeling (Neale et al., 1999). Preliminary sex-limitation analyses showed that constraining the prevalence of object attachment to be equal for girls and boys, setting the genetic correlation between opposite-sex twin pairs at 0.50 as for SSDZ twins, and equating genetic and environmental estimates for boys and girls did not worsen model fit (**Table 2**). We therefore estimated a single set of genetic and environmental effects with all combinations of male and female MZ and DZ twins.

**Table 2:** Tests for sex differences.

Model	Model fit				Difference from saturated model			
	*χ*^2^	DF	*p*	AIC	RMSEA	Δ*χ*^2^	DF	*p*
Saturated model (sex differences allowed)	9.937	11	0.536	-12.063	0.00			
Equate prevalences for males and females	10.208	12	0.598	-13.792	0.00	0.271	1	0.603
Constraining the genetic correlation between opposite-sex twins to equal 0.50 as for DZ same-sex twins	10.208	13	0.677	-15.792	0.00	0.271	2	0.873
Constraining genetic and environmental variance component estimates as identical for boys and girls	10.463	15	0.79	-19.537	0.00	0.526	4	0.971

*AIC, Akaike’s information criterion; RMSEA, root-mean-square error of approximation.*

A significant genetic effect was found, accounting for 48% of the variance (95% confidence interval [CI]: 0.26–0.69), as well as a significant and large shared environment effect accounting for 48% of the variance (CI: 0.29–0.65). The non-shared environment effect was estimated at 5% only (CI: 0.01–0.13). Model fit was excellent, as indicated by several fit indices: *χ*^2^(15) = 10.46, *ns*, Akaike information criterion (AIC) = -19.54, and root mean square error of approximation (RMSEA) = 0.000. None of the model components could be dropped without significantly reducing the model fit.

### CHILDCARE DESCRIPTIVES

Daycare information was available for 1054 twin pairs. Since the vast majority of the children in this sample were enrolled in childcare at age 3 (98%), the following analyses focus on these children only. Children spent between 4 and 10.5 h per day at daycare (*M* = 7.27 h, SD = 1.34); group size ranged from 3 to 40 (*M* = 23.48 children, SD = 8.05), and mean child-to-teacher ratio was 8.5 (SD = 4.00).

### CHILDCARE QUALITY AND OBJECT ATTACHMENT

Quality indicators of daycare such as group size [*t*(965) = 0.42, ns], number of daycare providers [*t*(994) = 1.04, ns], and child-to-teacher ratio [*t*(948) = 0.66, ns] were unrelated to children’s object attachment. Results remained non-significant when comparisons included only children who were not at neonatal risk as defined by prematurity, low birth weight, or admission to NICU.

### CHILDCARE QUANTITY AND OBJECT ATTACHMENT

**FIGURE 1:**
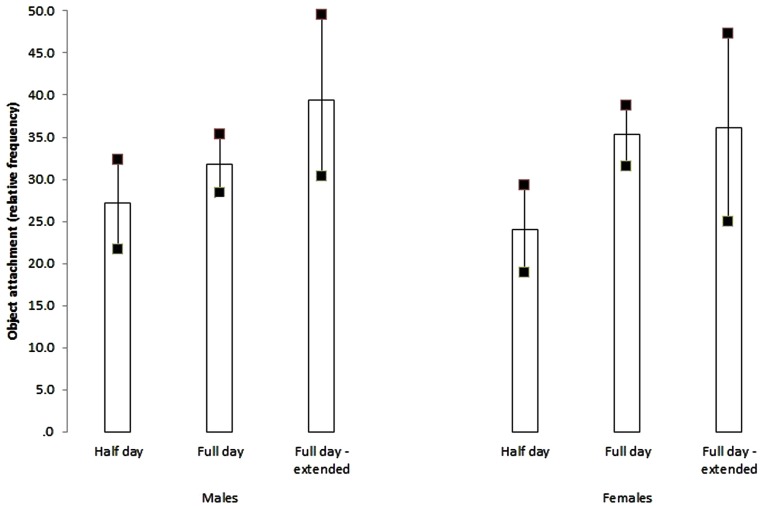
**Prevalence (and 95% confidence intervals) of children’s object attachment by time spent daily in non-maternal childcare**.

According to reports of the time per day that children spent at daycare we divided childcare quantity into half-day (staying at daycare until between noon and 1:30 pm, 26% of children) and full-day (daily stay until between 3 pm and 6:30 pm, 74% of children) and compared object attachment rates in the two groups. Among the children who stayed at daycare only half days, rates of object attachment were only 27.3%, whereas for children who regularly spent full days under organized care, object attachment reached significantly higher rates of 35.6%, *χ*^2^(1) = 5.98, *p* = 0.01. When we further split full-day stay into full-day (up to 4:30 pm, 65%), and full-day-extended (until 5 pm or later, 9%), there was further differentiation between the groups, *χ*^2^(2) = 6.60, *p* < 0.04 (see **Figure 1**). Thus, childcare quantity was found to relate to children’s tendency to be object-attached. Similar rates were found among the three daycare groups when children with known neonatal risk were excluded (27.8%, 37%, and 41.2%, respectively).

### DOES CHILDCARE QUANTITY ACCOUNT FOR VARIATION IN CHILDREN’S OBJECT ATTACHMENT?

In order to test whether childcare quantity explains some of the environmental variation in object attachment we used Jaffee et al.’s (2002) model to decompose the variance in object attachment into genetic, shared environmental, and non-shared environmental factors, plus childcare quantity (as a measured variable). This model estimates the variance as potentially composed of four effects: the regular effects estimated in a twin model (genetic, shared environment, and non-shared environment), but in addition, the effect of a variable that does not vary between twins in the same pair. This last effect is estimated based on the association between the phenotype (here, object attachment) and the environmental variable (here, childcare quantity.) [It is important to test that the environmental effect does not actually reflect a genetic effect influencing the tendency to send children to daycare (see Turkheimer et al., 2005); preliminary analyses with a model by Price and Jaffee (2008) showed no such evidence for a gene-environment correlation (rGE) involved in daycare quantity].

Twin pairs were fully concordant on childcare quantity. Results indicate that childcare quantity (half-day vs. full-day) significantly (*p* < 0.05) predicted use of an attachment object, accounting for 2.2% of the shared environmental effect.

## DISCUSSION

Young children’s attachment toward inanimate objects is a well-known, yet under-studied, developmental phenomenon. Our results point to the importance of considering both environmental as well as genetic influences on children’s object attachment behaviors. Close to half of the variation in object attachment was attributed to the effects of the shared environment (48%). Furthermore, findings point to a specific shared environmental influence on this behavior; children who spent many hours in center-based childcare at the age of 3 were more likely to develop attachments to inanimate objects relative to children who spent only half-days in daycare. Much has been said about the implications that the shift from home-care to non-maternal group-care of young children has for their early development and adjustment. Object attachment might be one way by which young children cope with spending many hours at daycare on a regular basis. Findings also shed some light on this little understood behavior and its relation to children’s rearing environment.

Having identified a link between childcare and object attachment, it is of further interest to better understand the developmental implications of a child having an attachment object for childhood and later on. It is generally considered to be part of normal development (Winnicott, 1953; Bowlby, 1969/1982), and the ability to rely on an object that is accessible and manipulated by the child is thought to have a facilitative influence under anxiety-evoking situations (Passman, 1976). Most published studies show no association between object attachment and concurrent behavioral disturbances (e.g., Garrison and Earls, 1982), with some studies even showing a positive association between soft object attachment and attachment security to mothers (Lehman et al., 1992; although see van Ijzendoorn et al., 1983 for no such association). Still, it is by no means an essential developmental step, as many children do not develop such emotional dependencies toward non-social objects, and there may be some less optimal long-term associations related with this behavior (e.g., Cohen and Clark, 1984; Markt and Johnson, 1993). For example, the presence of strong and persistent attachments to objects during childhood was linked with higher excitability, restlessness, and impatience among college-age young adults (Cohen and Clark, 1984). Such findings may suggest a common underlying cause for object use and emotion and stress regulation patterns. Further study is needed to test such hypotheses.

On a related point, we found a substantial portion of the variation in object attachment to be attributed to genetic factors. Over recent decades, behavior genetic research has demonstrated that genetic variability serves as a foundation for individual differences in many complex traits (Plomin et al., 2001). Current findings suggest that object attachment is a phenotypic marker of some underlying genetically driven mechanisms, perhaps related to stress reactivity/regulation. Further studies are needed to clarify the meaning of this genetic basis; for example, it would be interesting to test whether object attachment in children is tied to biological functions (i.e., cortisol production).

Previous findings suggested that object attachment is more prevalent among children in middle and upper socioeconomic background, and is related to mothers’ educational level (Gaddini and Gaddini, 1970; Litt, 1986). Our findings are more consistent with studies which do not indicate such associations (Boniface and Graham, 1979; Passman and Halonen, 1979), and thus strengthen the understanding that it is likely childrearing practices involving frequent separation from parents rather than family background that are related to children’s dependence on non-social objects for a sense of security.

This study has a few limitations. First, findings are based on maternal reports. Richer information could be achieved by observations of children’s behavior and ways in which attachment to objects is expressed, especially in the context of daycare setting. That said, the use of surveys enabled us to reach hundreds of twin pairs across the country. Second, the sample is comprised of twins only, who may not be fully representative of all children of the same age in terms of the measured variables. It is possible that object attachment functions differently for twins as compared to singletons given that twins are mostly present in each other’s lives early on, and the special relationship between twin siblings (e.g., Fortuna et al., 2010).

In sum, it is our hope that this report contributes to the vast literature on early childcare in relation to child development by identifying a novel association linking daycare quantity and young children’s attachment to inanimate objects. In addition, this paper adds to the gradually accumulating appreciation for the origins of this intriguing behavior in young children, which occupies many parents and other care providers, though clearly much is left to be explored on this topic to be translated into practice toward optimal development of children.

## Conflict of Interest Statement

The authors declare that the research was conducted in the absence of any commercial or financial relationships that could be construed as a potential conflict of interest.
